# In Gouty Rheumatism

**Published:** 1894-02

**Authors:** 


IN GOUTY RHEUMATISM.—It appears to be quite the fashion to represent all high-livers as suffering from the gout. But it has been our observation that there are certain rheumatic conditions which are more or less of a gouty nature, so that no other term describes the condition quite as well as that of gouty rheumatism. But more than this, we have noticed such a condition to give rise to a most obstinate throat affection, a condition of things first brought to our attention by Dr. Ingals, of Chicago. In all such cases we are in the habit of prescribing Henry's Tri-Iodides, a mixture composed of those ingredients most suitable for such conditions. We notice that a number of physicians report cases of gout and rheumatism relieved by this preparation when the usual remedies had failed to bring relief. We are very glad to add here that after an acquaintance with Mr. F. A. Henry for a long time we know the high plane upon which he does business. He is very careful to confine the distribution of his products through the regular channels of the medical profession, not attempting and not desiring in any way to deal with the laity.—*Food.*

